# Mass Lead Poisoning in Dakar: Battery Recycling Exacts a Heavy Toll

**Published:** 2009-10

**Authors:** Carol Potera

**Affiliations:** **Carol Potera**, based in Montana, has written for *EHP* since 1996. She also writes for *Microbe*, *Genetic Engineering News*, and the *American Journal of Nursing*

In a neighborhood of Dakar, Senegal, 18 children died from an aggressive central nervous system disease between November 2007 and March 2008. Experts from the World Health Organization and local health authorities were called in to investigate the deaths, but cultural prohibitions preempted autopsies of the children. So the researchers examined 32 of the children’s siblings and 23 of the siblings’ mothers along with 18 unrelated local children and 8 unrelated adults. They concluded that the cause of death likely was encephalopathy resulting from severe lead poisoning **[*EHP*** **117:1535–1540; Haefliger et al.]**. The source of the lead, in turn, was determined to be contamination resulting from the reclamation of used lead‐acid batteries, a lucrative business in developing countries that often is performed in the open with few pollution controls.

Since 1995, local people had broken apart batteries from vehicles and appliances and sorted the components in an open sandy area of the neighbhorhood. They sifted through the sand for scraps of valuable lead to sell, even carrying sacks of contaminated sand into their homes. People were probably exposed by inhaling and ingesting lead dust, with children particularly exposed through hand‐to‐mouth activity and eating the contaminated soil.

The developing nervous system of children is particularly vulnerable to the toxic effects of lead. Blood lead concentrations as low as 10 μg/dL are known to impair neurologic development, resulting in permanent intellectual impairment. However, recent evidence suggests there may be no safe threshold of exposure.

Among the 50 children tested, blood lead levels ranged from 39.8 to 613.9 μg/dL. Seventeen of the 50 children showed neuropsychiatric symptoms including convulsions, irritability, and aggression, and 21 showed gastrointestinal symptoms such as anorexia and vomiting. Adult blood lead levels ranged from 32.5 to 98.9 μg/dL, and their most commonly reported symptom was gastrointestinal upset.

Recycling activity reportedly ended by March 2008 following a public awareness campaign, and the neighborhood soil was partially remediated. Nevertheless, lead concentrations measured in the sandy work area after this time still reached 209,000 mg/kg, and levels inside homes reached 14,000 mg/kg. The U. S. Department of Housing and Urban Development sets 400 mg/kg as for the standard for lead in bare soil in children’s play areas (there is no comparable standard in Senegal).

The lead poisoning in this study was severe enough to catch the attention of health experts, but the global incidence of lead poisoning from battery recycling is unknown. The authors believe many cases go unaddressed in developing countries because local authorities lack resources to recognize, diagnose, and manage lead toxicity. However, they write, lead poisoning can be prevented through measures such as public education and the implementation and enforcement of lead recycling guidelines.

## Figures and Tables

**Figure:**
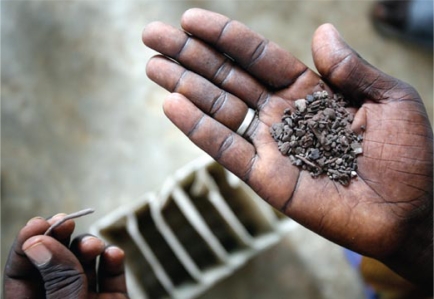
The father of a fatally lead-poisoned Dakar child holds lead extracted from a spent car battery, September 2008

